# Supplementary material for: Cathelicidins prime platelets to mediate arterial thrombosis and tissue inflammation
Source: Nat Commun. 2018 Apr 18;9:1523. doi: 10.1038/s41467-018-03925-2 (PMC5906636; doi:10.1038/s41467-018-03925-2)
Supplement: Supplementary file 1 — Supplementary Information [file 41467_2018_3925_MOESM1_ESM.pdf]

Supplementary Figure 1

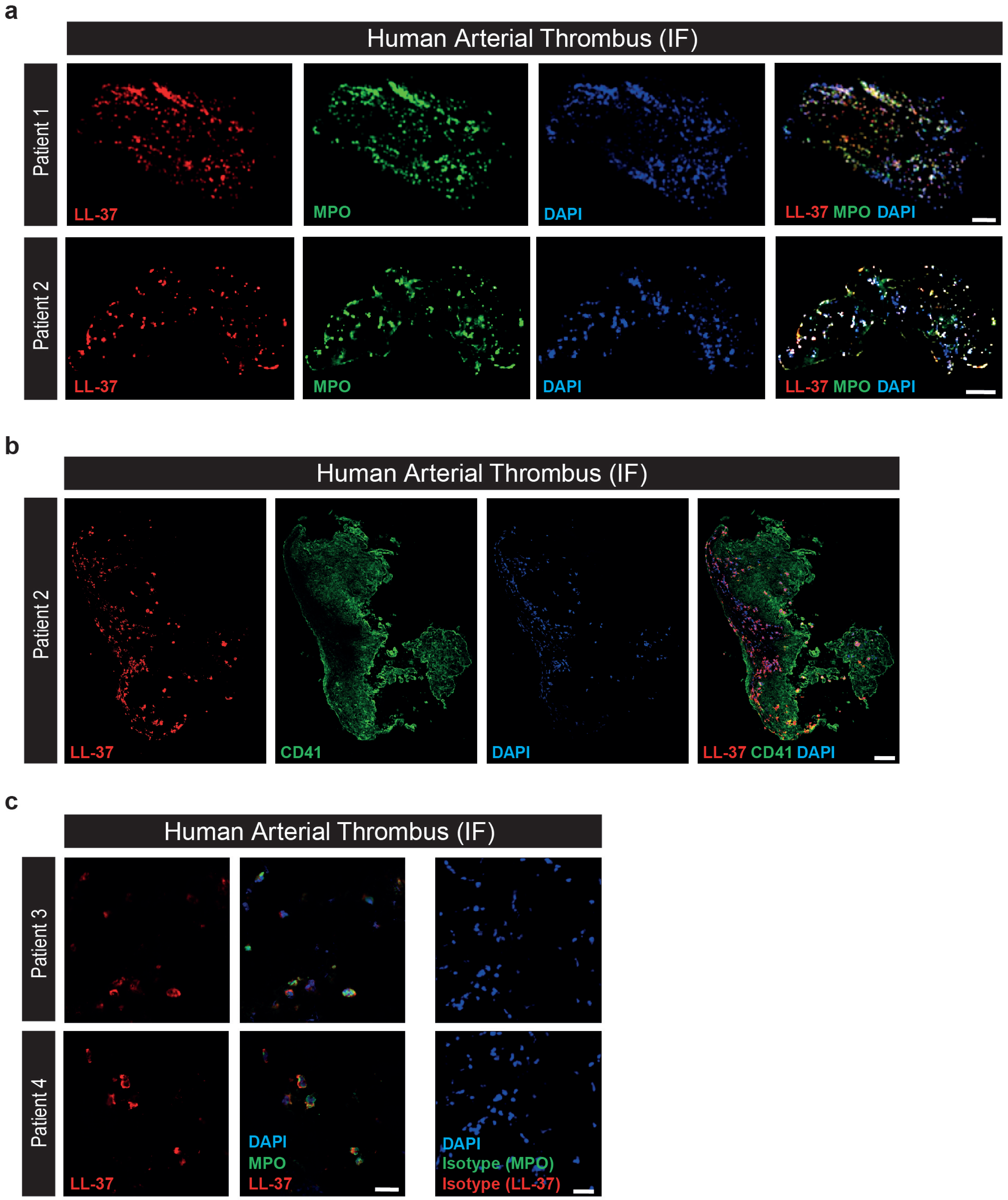

Supplementary Figure 1: LL-37 immunofluorescence in human arterial thrombi

(a-c) Immunofluorescence images of coronary artery thrombi isolated from patients with acute myocardial infarction. (a) Cryosections were stained for LL-37 (red), neutrophil myeloperoxidase (MPO, green) and DAPI (nuclear stain, blue). Bars, 200 µm. (b) Cryosections were stained for LL-37 (red), platelets (CD41, green) and DAPI (nuclear stain, blue). Bar, 200 µm. (c) Cryosections from the same patient were stained for LL-37 (red) and MPO (green) or respective isotype control antibodies (LL-37 control in red, MPO control in green), and DAPI (blue). Bar is 10 µm for left and middle images, and 50 µm for right images.

Supplementary Figure 2

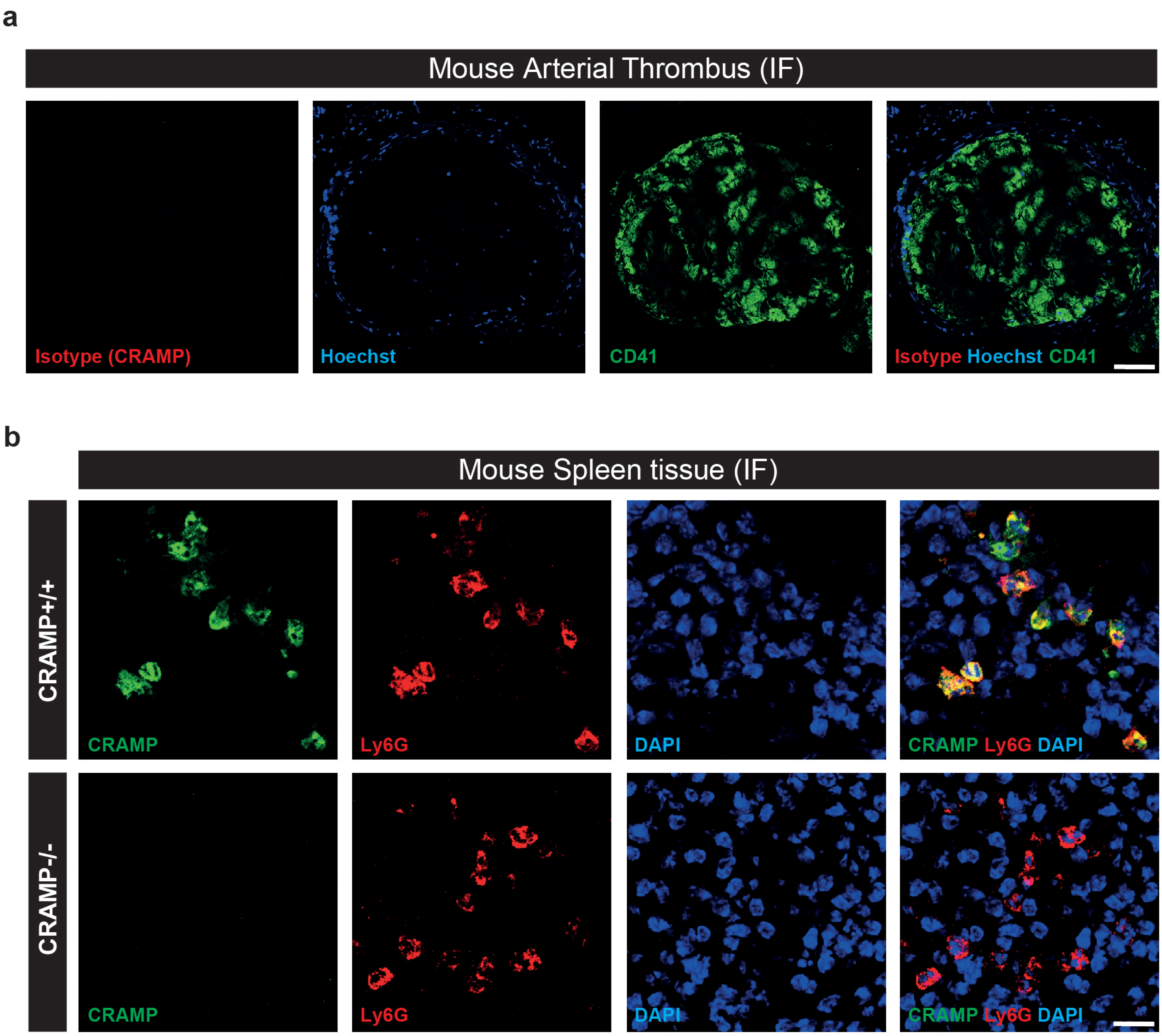

Supplementary Figure 2: CRAMP immunofluorescence control stainings
(a) Cryosections of ferric chloride-induced mouse carotid artery thrombi were stained with isotype control antibody for CRAMP (red), platelets (CD41, green) and Hoechst (nuclear stain, blue). Bar, 100 µm. (b) Specificity of the CRAMP-antibody was confirmed in cryosections of mouse spleen tissue of wildtype and *CRAMP*$^{-/-}$ mice. CRAMP-antibody (green), Ly6G (red) and DAPI (nuclear stain, blue). Bar, 20 µm.

Supplementary Figure 3

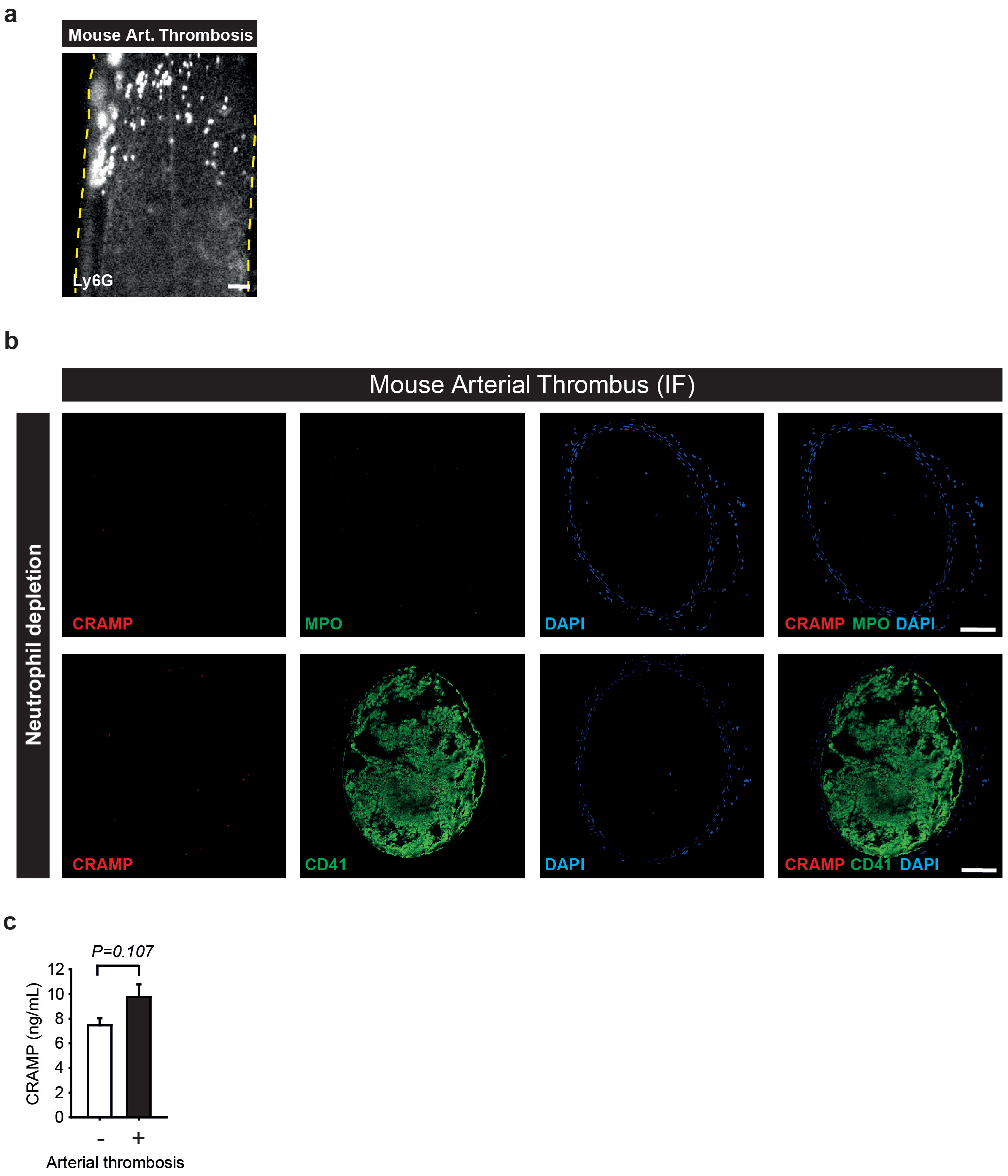

Supplementary Figure 3: Neutrophil recruitment in mouse arterial thrombosis
(a) Neutrophil recruitment after carotid artery injury induced by temporary mechanical ligation. Neutrophils were labeled in vivo using a fluorescent Ly-6G antibody (PE). Representative intravital microscopy image 10 minutes after induction of injury is shown. Bar, 50 µm. (b) Antibody-mediated neutrophil-depletion in mouse $FeCl_3$-induced carotid artery thrombosis. Cryosections were stained for CRAMP (red) and DAPI (nuclear stain, blue), and myeloperoxidase (MPO, green, upper row) or platelets (CD41, green, lower row). Bars, 100 µm. (c) CRAMP plasma levels two hours after thrombus induction with ferric chloride (n=4-5). Graph shows mean and SEM. *P*-values were determined by unpaired t-test.

Supplementary Figure 4

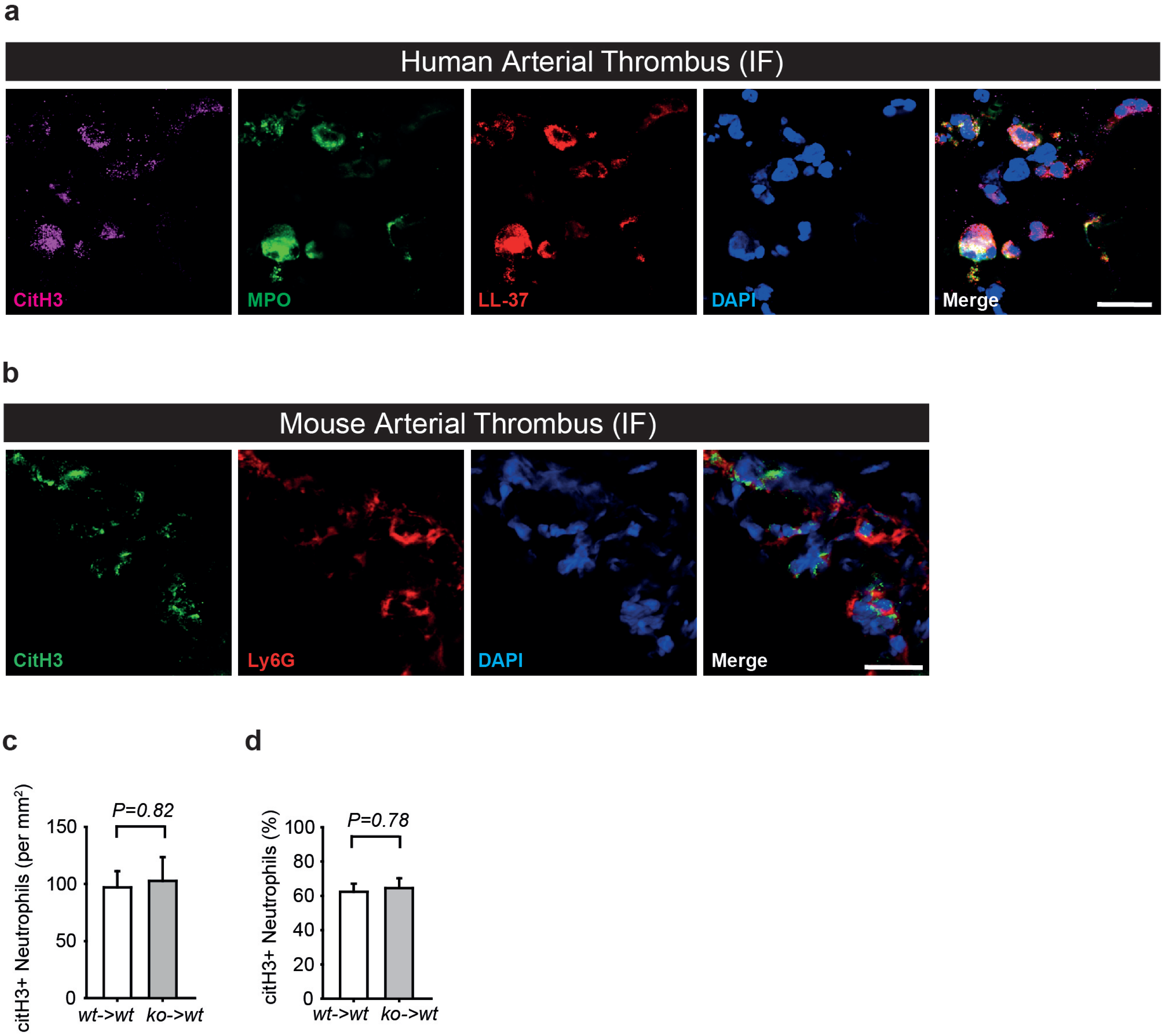

Supplementary Figure 4: Neutrophil activation in human and mouse arterial thrombosis
(a-d) Analysis of neutrophil citrullinated histone H3 (citH3) staining in human (a) and mouse (b-d) arterial thrombi. (a) Immunofluorescence images of a coronary artery thrombus isolated from a patient with acute myocardial infarction. Cryosections were stained for citrullinated histone H3 (citH3, magenta), myeloperoxidase (MPO, green), LL-37 (red) and DAPI (nuclear stain, blue). Bar, 10 µm. (b) Immunofluorescence images of a mouse $FeCl_3$-induced carotid artery thrombus. Cryosections were stained for citH3 (green), Ly6G (red) and DAPI (nuclear stain, blue). Bar, 10 µm. (c-d) Quantification of citH3 staining in Ly6G+ neutrophils in wildtype (wt->wt) and *CRAMP-/-* (ko->wt) bone marrow chimeric mice (n=8). Graphs show mean and SEM. *P*-values were determined by unpaired t-test.

Supplementary Figure 5

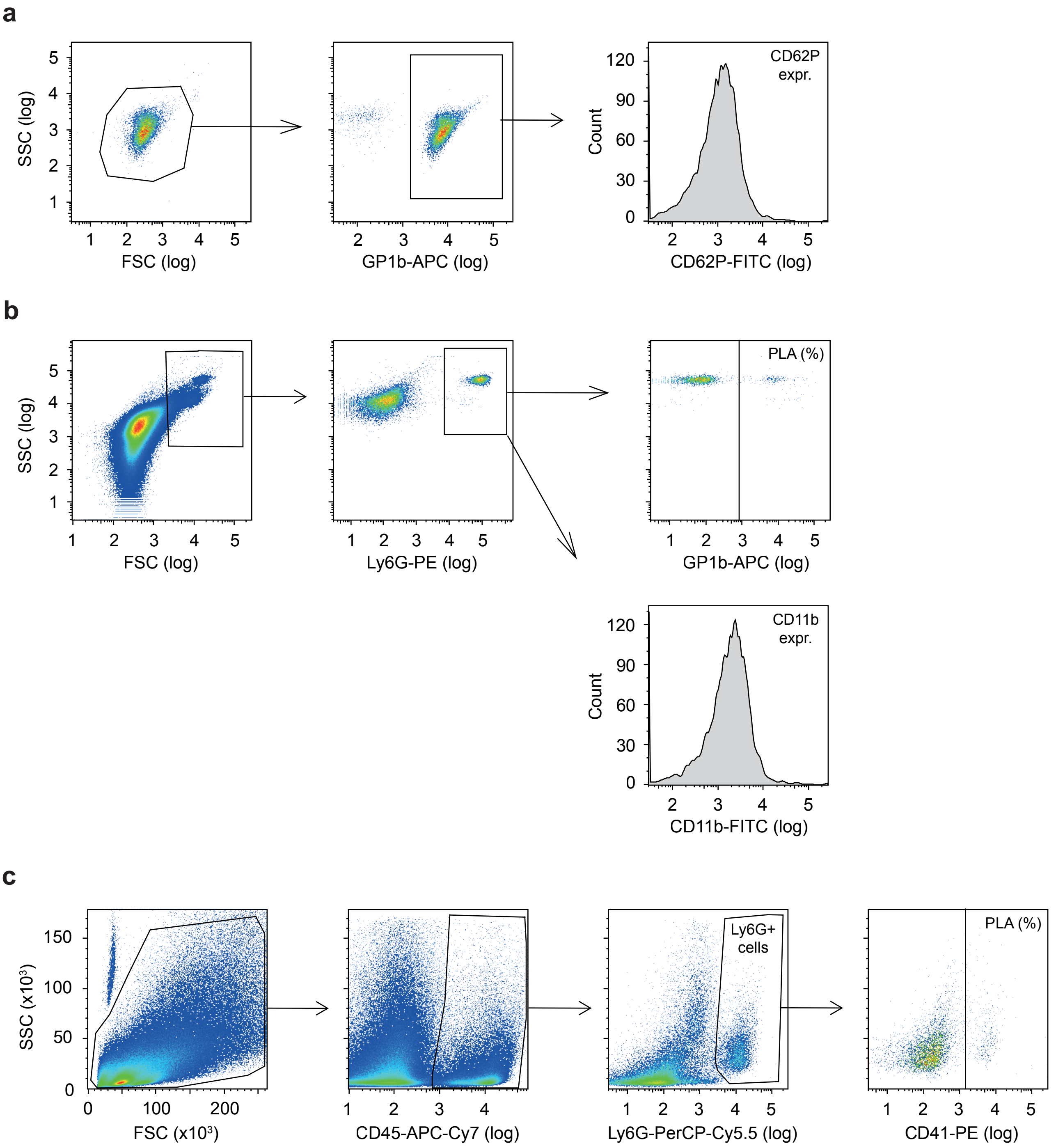

Supplementary Figure 5: Flow cytometry gating strategy

(a) Gating strategy was used to measure expression of surface molecules in human or mouse platelets (Fig. 3a,c-i, Fig. 4a-c,e-k, Fig. 7o-r and Suppl. Fig. 7; exemplary demonstrated for P-Selectin surface expression) and to assess binding of fluorescently labeled cathelicidins to human or mouse platelets (Fig. 1c). (b) Gating strategy was used to measure human or mouse platelet-neutrophil aggregates (PLA; Fig. 5a,b,i,j, Fig. 6c, Fig. 7m) and to measure neutrophil activation (Fig. 5c-e; exemplary demonstrated for surface expression of CD11b). (c) Gating strategy was used to assess alveolar and interstitial (including pulmonary vasculature) neutrophil infiltration (Fig. 7d,e,g,h) and platelet-neutrophil aggregates in the pulmonary vasculature (Fig. 7n) in acute lung injury.

Supplementary Figure 6

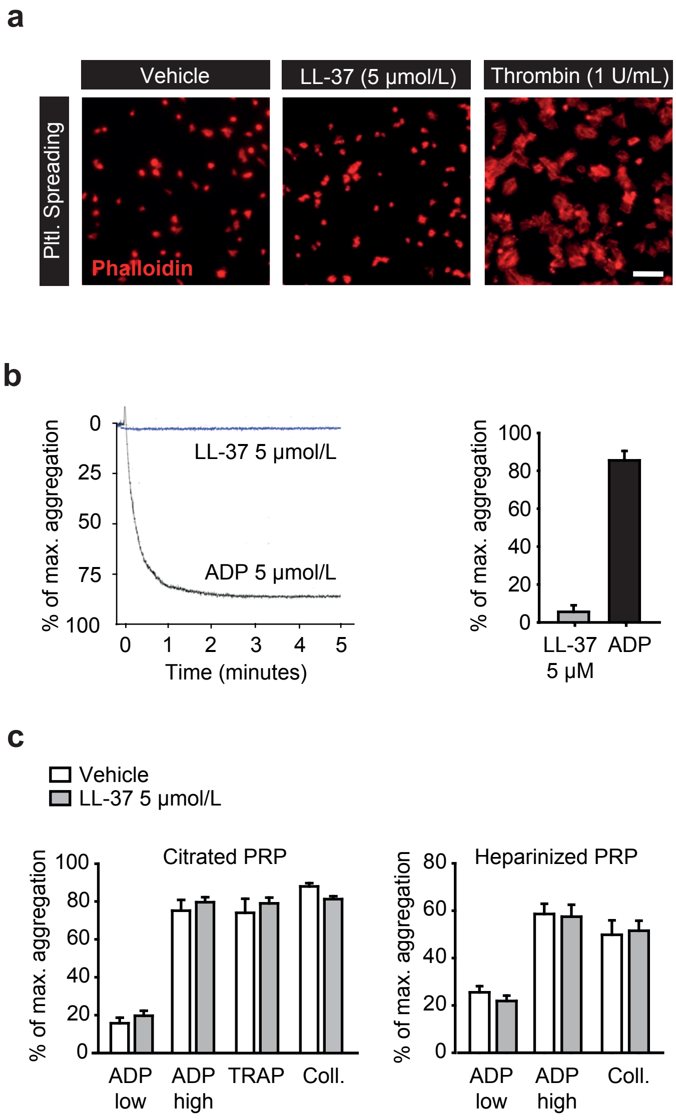

Supplementary Figure 6: Platelet spreading and aggregation

(a-c) Platelet spreading and aggregation. (a) Platelet spreading upon incubation of washed human platelets with LL-37. Visualizing of the actin-cytoskeleton with Alexa546-phalloidin (red). Thrombin was used as positive control (images are representative of 3 independent experiments; bar, 10 µm). (b) Aggregation of human platelets in platelet-rich plasma (PRP) upon addition of LL-37 (n=3). ADP served as positive control. (c) ADP-, TRAP (thrombin receptor activator) or collagen-induced platelet aggregation after preincubation with LL-37 (5 µmol/L) in citrate (n=6-8; left) or heparinized (n=5-6; right) human PRP. Graphs show mean and SEM.

Supplementary Figure 7

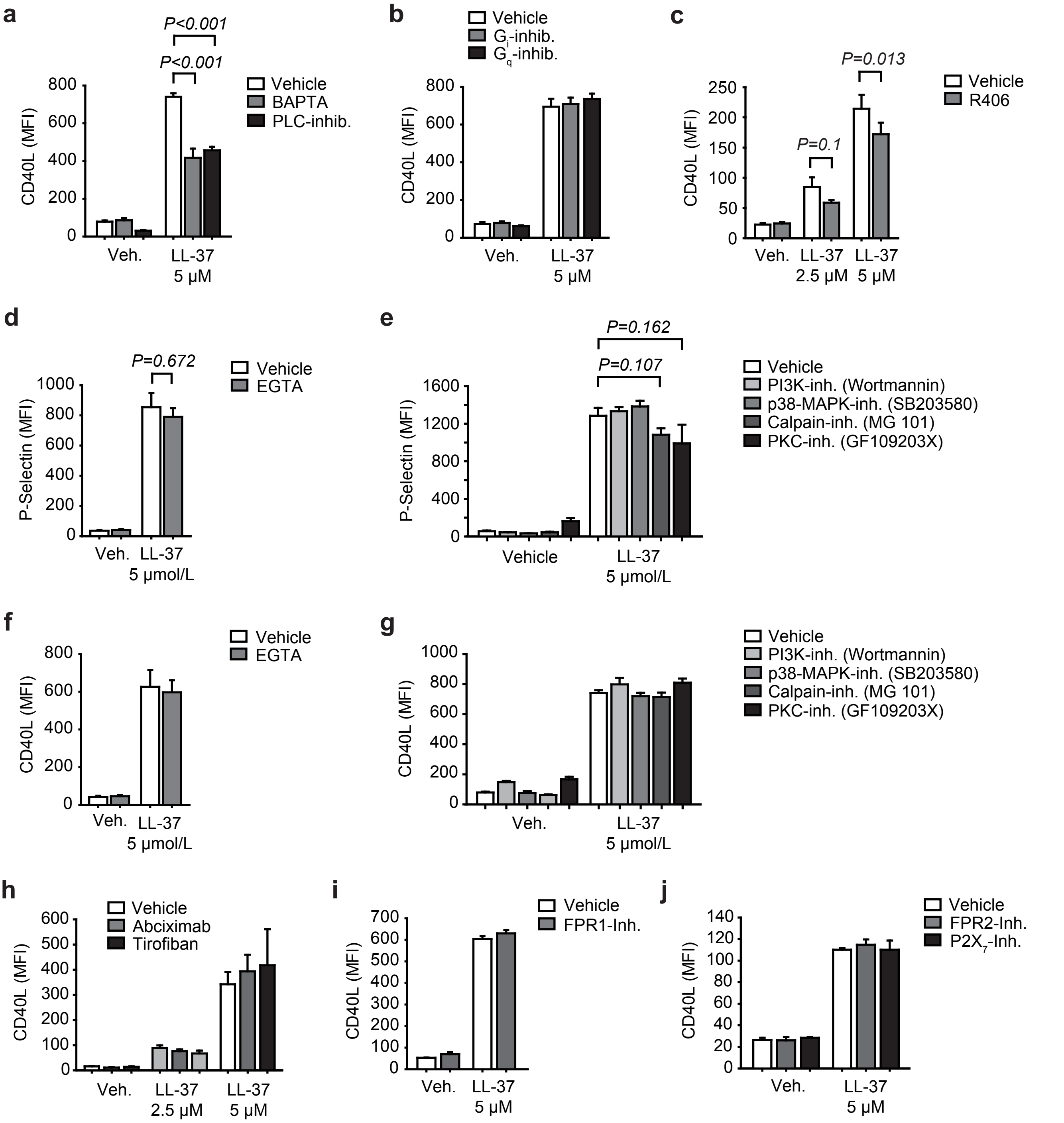

Supplementary Figure 7: LL-37 activates human platelets

(a-j) Flow cytometry analysis of LL-37 effects on isolated human platelets. (a-c) Effect of LL-37 on platelet CD40L surface expression after (a) inhibition of intracellular calcium release (BAPTA) or phospholipase C (U-73122; n=5 and n=6, respectively), (b) inhibition of G-Protein signaling (Pertussis toxin or cholera toxin, n=4), (c) inhibition of tyrosine Syk (R406; n=5). (d,e) Effect of LL-37 on platelet P-Selectin surface expression after (d) quenching of extracellular calcium with EGTA (n=4), (e) inhibition of PI3 kinase, p38-MAPK, Calpain or protein-kinase C (PKC) with the inhibitors Wortmannin, SB203580, MG 101 or GF109203X, respectively (n=4, each). (f,g) Effect of LL-37 on platelet CD40L surface expression after (f) quenching of extracellular calcium with EGTA (n=4), (g) inhibition of PI3 kinase, p38-MAPK, Calpain or protein-kinase C (PKC) with the inhibitors Wortmannin, SB203580, MG 101 or GF109203X, respectively (n=4, each). (h-j) Effect of LL-37 on platelet CD40L surface expression after (h) inhibition of the glycoprotein (GP)IIb/IIIa receptor with a blocking antibody (Abciximab) or inhibitor (Tirofiban; n=4), (i) inhibition of the formyl-peptid-receptor 1 (FPR1) with Boc-MLF 10, (j) inhibition of formyl-peptide-receptor 2 (FPR2) with WRW4 or the purinergic P2X7-receptor with A438079 (n=4, each). Graphs show mean and SEM. *P*-values were determined by unpaired (a,e) or paired t-test (c,d).

Supplementary Figure 8

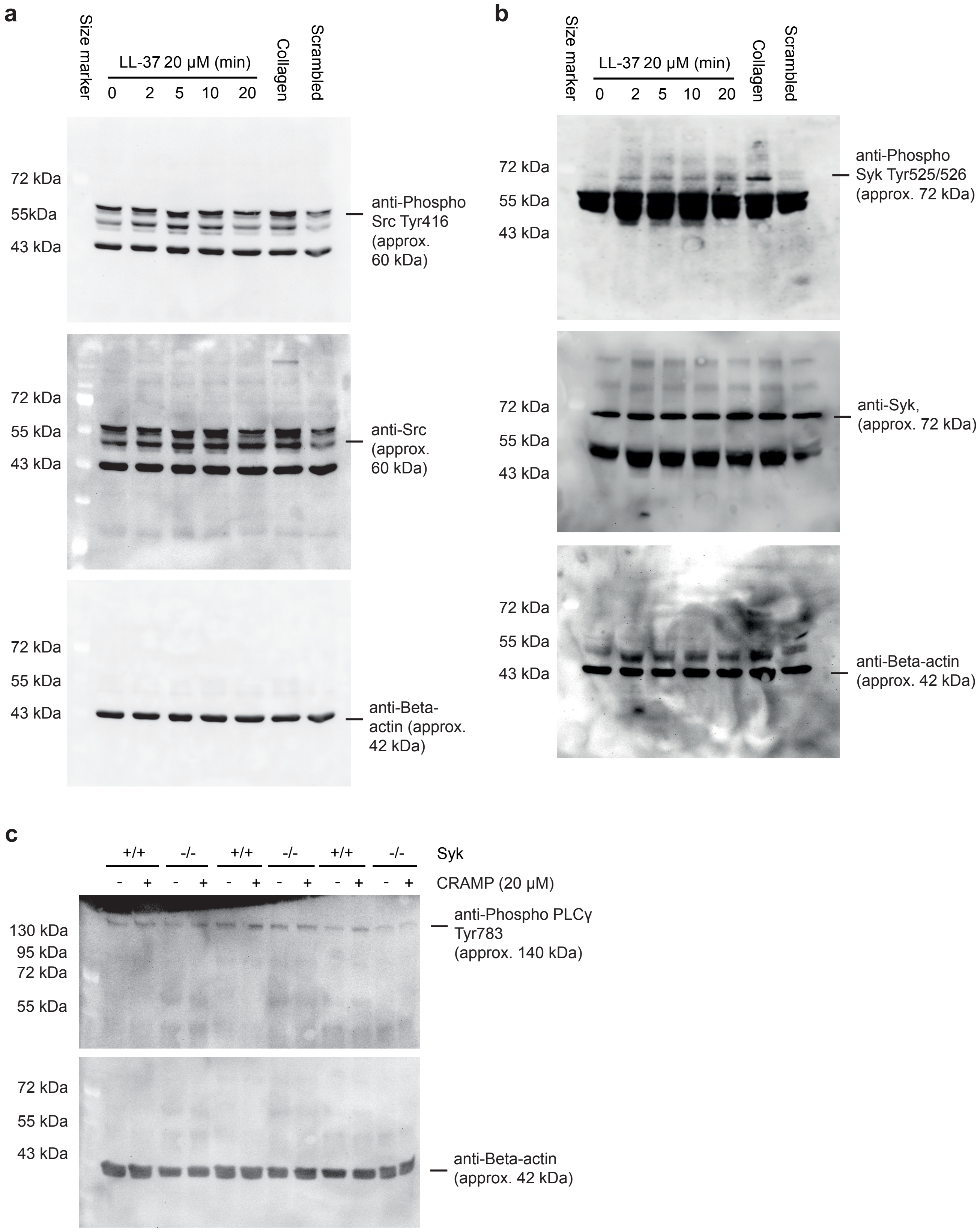

Supplementary Figure 8: Full western blots

(a-c) Full western blots of blot portions shown in Fig. 4d (a,b) and 4l (c) with respective size markers. (a) Western blot of human platelet lysates for phospho-Src, Src and beta-actin (loading control). (b) Western blot of human platelet lysates for phospho-Syk, Syk and beta-actin (loading control). (c) Western blot of Syk+/+ or Syk-/- mouse platelets for phospho-phospholipase Cγ and beta-actin (loading control).

Supplementary Figure 9

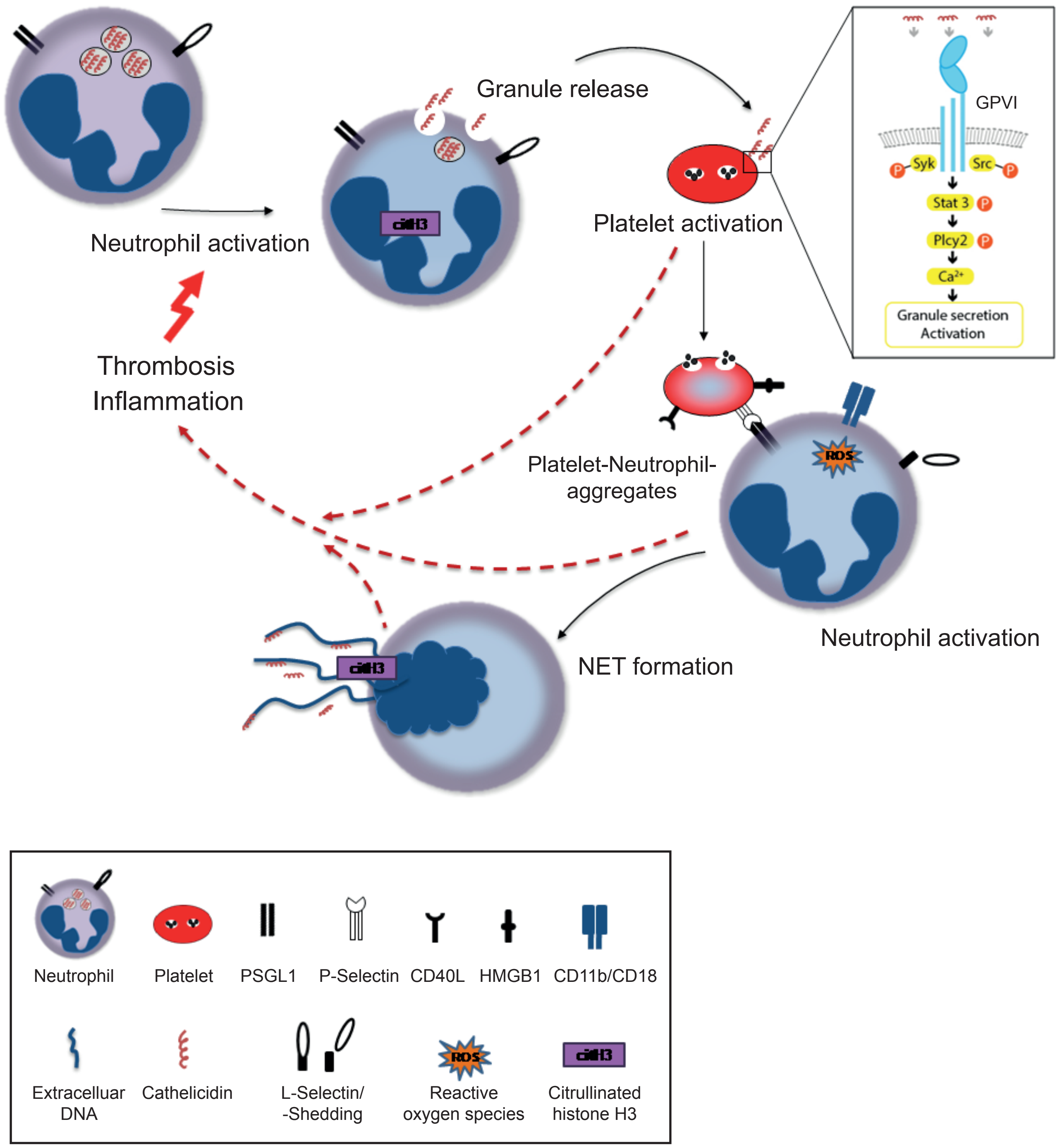

Supplementary Figure 9: Schematic of Cathelicidin-induced platelet activation
Schematic of Cathelicidin-induced platelet activation. Activated neutrophils release cathelicidins (human LL-37 or mouse CRAMP), which induce platelet activation as indicated by surface expression of P-Selectin, CD40L and HMGB1. Cathelicidin signaling involves the platelet GPVI receptor complex and downstream signaling through protein-tyrosine kinases Src/Syk and phospholipase C. Cathelicidin-activated platelets bind neutrophils in a P-Selectin dependent manner to form platelet-neutrophil aggregates and induce neutrophil activation (ROS production, CD11b surface expression, L-Selectin shedding) and NET formation. These effects may contribute to arterial thrombosis, neutrophil extravasation at sites of tissue inflammation and acute lung injury *in vivo*.

Supplementary Table 1

	CRAMP$^{+/+}$ BM	*CRAMP*$^{-/-}$ BM	*P-value*
HGB (g/dL)	14.1 [13.6, 15,0]	14.1 [13.9, 15,2]	*0.869*
RET (%)	4.0 [3.9, 4.7]	5.4 [4.4, 6.4]	*0.229*
Plt (10^3/µL)	831 [683, 894]	703 [642, 780]	*0.189*
WBC (10^3/µL)	8.7 [7.2, 13.3]	8.7 [6.2, 11.7]	*0.473*
NEUT (%)	33 [16, 49]	20 [14, 27]	*0.193*
LYMPH (%)	64 [47, 82]	76 [71, 84]	*0.199*
MONO (%)	2.1 [1.8, 3.8]	1.8 [0.9, 2.5]	*0.212*
EOSINO (%)	0.8 [0.5, 1.8]	1.4 [1.2, 2.0]	*0.404*

Supplementary Table 1: Differential blood cell counts in bone marrow chimeric mice
Differential blood cell counts in *Cramp*$^{+/+}$ and *Cramp*$^{-/-}$ bone marrow chimeric mice. Data are shown as median [Q1, Q3], n=9. *P*-values were determined by unpaired t-test or Mann-Whitney U test.

Supplementary Table 2

	CRAMP$^{+/+}$ BM	*CRAMP*$^{-/-}$ BM	*P-value*
CD41 [MFI]	13790+/-299	14396+/-693	*0.172*
CD61 [MFI]	6372+/-235	6492+/-181	*0.695*
CD42b [MFI]	13325+/-587	14928+/-699	*0.110*
GPVI [MFI]	10811+/-138	10182+/-346	*0.180*
GPIX [MFI]	608+/-44	545+/-45	*0.339*
CD62P [MFI]	1330+/-58	1324+/-32	*0.934*
act. GPIIbIIIa [MFI]	2054+/-126	2245+/-78	*0.225*
PS [MFI]	121+/-13	130+/-18	*0.679*

Supplementary Table 2: Baseline platelet surface molecules in bone marrow chimeric mice
Baseline platelet surface molecules in *Cramp*$^{+/+}$ and *Cramp*$^{-/-}$ bone marrow chimeric mice. Identical flow cytometry settings were used throughout the experiments. Data are shown as mean and SEM, n=6. *P* values were determined by unpaired t-test or Mann-Whitney U test. MFI: Mean fluorescence intensity. PS: Phosphatidylserine. GP: Glycoprotein.

Supplementary Table 3

	CRAMP$^{+/+}$	*CRAMP*$^{-/-}$	*P-value*
CD62P [MFI]	327+/-45	319+/-39	*0.892*
CD40L [MFI]	185+/-43	196+/-37	*0.394*
act. GPIIbIIIa [MFI]	164+/-22	156+/-20	*0.669*

Supplementary Table 3: Baseline platelet activation markers in CRAMP knockout mice
Baseline platelet activation markers in *Cramp*$^{+/+}$ and *Cramp*$^{-/-}$ mice. Identical flow cytometry settings were used throughout the experiments. Data are shown as mean and SEM, n=6. *P*-values were determined by unpaired t-test or Mann-Whitney U test. MFI: Mean fluorescence intensity. GP: Glycoprotein.
