# Supplementary material for: Cathelicidins prime platelets to mediate arterial thrombosis and tissue inflammation
Source: Nat Commun. 2018 Apr 18;9:1523. doi: 10.1038/s41467-018-03925-2 (PMC5906636; doi:10.1038/s41467-018-03925-2)
Supplement: Supplementary file 3 — Description of Additional Supplementary Files [file 41467_2018_3925_MOESM3_ESM.pdf]

Description of Additional Supplementary Files

Supplementary Movie 1: CRAMP binding in arterial thrombosis *in vivo*

Time-lapse video of ferric chloride induced thrombus formation in the mouse carotid artery imaged by intravital epifluorescence microscopy. 5-FAM-labeled CRAMP was injected into wildtype mice before induction of ferric chloride injury. Platelets were labeled *in vivo* using a DyLight649-labeled non-blocking GPIb antibody. 5-FAM-labeled CRAMP (green) associated with platelets (GPIb, red) in the forming thrombus. Video shows thrombus formation 4 to 7 minutes after injury. Bar, 200µm.

Supplementary Movie 2: Scrambled control peptide in arterial thrombosis *in vivo*

Time-lapse video of ferric chloride induced thrombus formation in the mouse carotid artery imaged by intravital epifluorescence microscopy. 5-FAM-labeled scrambled control was injected into wildtype mice before induction of ferric chloride injury. Platelets were labeled *in vivo* using a DyLight649-labeled non-blocking GPIb antibody. 5-FAM-labeled control peptide (green) showed not binding to platelets (GPIb, red) in the forming thrombus. Video shows thrombus formation 4 to 7 minutes after injury. Bar, 200µm.

Supplementary Movie 3: Arterial thrombosis in wildtype BM chimeric mouse

Time-lapse video of thrombus formation in the mouse carotid artery. Injury was induced by ferric chloride in wildtype (wt->wt) BM chimeric mice and imaged by intravital epifluorescence microscopy. Blood cells were labeled *in vivo* with the fluorescent dye DiOC6 to visualize thrombus formation.

Supplementary Movie 4: Arterial thrombosis in CRAMP-deficient BM chimeric mouse

Time-lapse video of thrombus formation in the mouse carotid artery. Injury was induced by ferric chloride in CRAMP-deficient (ko->wt) BM chimeric mice and imaged by intravital epifluorescence microscopy. Blood cells were labeled *in vivo* with the fluorescent dye DiOC6 to visualize thrombus formation.

Supplementary Movie 5: Platelet-vessel wall interactions of vehicle treated platelets

Real-time video of platelet-vessel wall interactions in inflamed postcapillary venules of the mouse cremaster muscle. Rhodamine-labeled platelets were pre-treated with vehicle and transfused into wildtype recipient mice. Platelet tethering was imaged using intravital epifluorescence microscopy.

Supplementary Movie 6: Platelet-vessel wall interactions of CRAMP treated platelets

Real-time video of platelet-vessel wall interactions in inflamed postcapillary venules of the mouse cremaster muscle. Rhodamine-labeled platelets were pre-treated with CRAMP (20 µmol/L) and transfused into wildtype recipient mice. Platelet tethering was imaged using intravital epifluorescence microscopy.
